# Identification of wild-caught phlebotomine sand flies from Crete and Cyprus using DNA barcoding

**DOI:** 10.1186/s13071-018-2676-0

**Published:** 2018-02-17

**Authors:** Emmanouil Dokianakis, Nikolaos Tsirigotakis, Vasiliki Christodoulou, Nikos Poulakakis, Maria Antoniou

**Affiliations:** 1 Laboratory of Clinical Bacteriology, Parasitology, Zoonoses and Geographical Medicine, School of Medicine, University of Crete, Vassilika Vouton, P.O. Box 2208, GR-71003 Heraklion, Greece; 2 Biology Department, School of Sciences and Engineering, University of Crete, Vassilika Vouton, P.O. Box 2208, GR-70013 Heraklion, Crete Greece; 3 Natural History Museum of Crete, School of Sciences and Engineering, University of Crete, Knossos Av, P.O. Box 2208, GR-71409 Heraklion, Crete Greece

**Keywords:** Sand fly, *Phlebotomus*, *Sergentomyia*, Leishmaniasis, DNA barcoding, *cox*1, Crete, Cyprus, Molecular systematics

## Abstract

**Background:**

Phlebotomine sand flies (Diptera: Psychodidae) are vectors of *Leishmania* spp., protozoan parasites responsible for a group of neglected diseases called leishmaniases. Two sand fly genera, *Phlebotomus* and *Sergentomyia*, contain species that are present in the Mediterranean islands of Crete and Cyprus where the visceral (VL), cutaneous (CL) and canine (CanLei) leishmaniases are a public health concern. The risk of transmission of different *Leishmania* species can be studied in an area by monitoring their vectors. Sand fly species are traditionally identified using morphological characteristics but minute differences between individuals or populations could be overlooked leading to wrong epidemiological predictions. Molecular identification of these important vectors has become, therefore, an essential tool for research tasks concerning their geographical distribution which directly relates to leishmaniasis control efforts. DNA barcoding is a widely used molecular identification method for cataloguing animal species by sequencing a fragment of the mitochondrial gene encoding cytochrome oxidase I.

**Results:**

DNA barcoding was used to identify individuals of five sand fly species (*Phlebotomus papatasi*, *P. similis*, *P. killicki*, *Sergentomyia minuta*, *S. dentata*) circulating in the islands of Crete and Cyprus during the years 2011–2014. *Phlebotomus papatasi* is a known vector of zoonotic CL in the Middle East and it is found in both islands. *Phlebotomus similis* is the suspected vector of *Leishmania tropica* in Greece causing anthroponotic CL. *Phlebotomus killicki* was collected in Cyprus for the first time. *Sergentomyia minuta*, found to present intraspecific diversity, is discussed for its potential as a *Leishmania* vector. Molecular identification was consistent with the morphological identification. It successfully identified males and females, which is difficult when using only morphological characters. A phylogenetic tree was constructed based on the barcodes acquired, representing their genetic relationships along with other species from the area studied. All individuals identified were clustered according to their species and subgenus.

**Conclusions:**

Molecular identification of sand flies via DNA barcoding can accurately identify these medically important insects assisting traditional morphological tools, thus helping to assess their implication in *Leishmania* transmission.

## Background

Sand flies (Diptera: Psychodidae) are small (body length < 3 mm) haematophagous insects and vectors of the protozoan parasites *Leishmania* spp. In the Old World, sand flies of the genus *Phlebotomus* are involved in an epidemiological cycle where a female sand fly that feeds on a *Leishmania-*infected reservoir host can become infected and transmit the parasite while feeding on its next target [1]. *Leishmania* spp. can cause a group of diseases called leishmaniases which, in the Mediterranean Basin appear in two forms: visceral (VL) and cutaneous (CL). There are estimations that leishmaniases are responsible for 20 to 30 thousand deaths worldwide each year [2]. The known pattern of co-evolution between parasites and their vectors renders necessary the evolutionary study by means of species identification and the vectorial capacity of the sand flies in an area [3]. Monitoring sand flies in a region is, therefore, one of the most important steps towards predicting and controlling the disease.

Crete and Cyprus, in the southeastern Mediterranean, are foci of both forms of leishmaniasis. In Crete, cases of VL and canine leishmaniasis (CanLei) (both caused by *Leishmania infantum*) are quite frequent while cases of CL (caused by *Leishmania tropica*) are re-emerging [4]. *Phlebotomus* (*Larroussius*) *neglectus* is a proven vector of *L. infantum* which is abundant on the island [4–9] but the vector of *L. tropica* is not yet implicated. Moreover, *Phlebotomus* (*Paraphlebotomus*) *similis* has been found in CL foci in Crete and it is the suspected vector of *L. tropica*, like its sister species *Phlebotomus* (*Paraphlebotomus*) *sergenti* elsewhere, since their systematic relationship implies similar vectorial capacity [10–12]. In Cyprus, *Phlebotomus* (*Larroussius*) *tobbi* is the vector of *L. infantum* causing CanLei [13, 14]. *Leishmania donovani*, a recent introduction to Cyprus, causes both VL and CL and it was found in a CanLei case in a mixed infection with *L. infantum* [15, 16]. Vectorial capacity of sand flies circulating in Cyprus and their role in the *L. donovani* transmission cycle is yet to be determined [14]. Sand flies of the genus *Sergentomyia* are present throughout Greece [17] and Cyprus [14, 18]. *Sergentomyia* (*Sergentomyia*) *minuta* is the predominant species found in the Mediterranean having a doubtful taxonomic status presenting high levels of intraspecific diversity correlated with geographical distribution [19]. Genus *Sergentomyia* is not deeply studied for its implication in the transmission of *Leishmania* but its species appear capable of feeding on rodents [20].

It is evident that proper identification of sand flies in an area can help assess the risk of spread of leishmaniasis. Although it is quite demanding, morphological identification is the traditional method that sand fly taxonomists use. It requires careful preparation of specimens after a field trip and a high degree of expertise [21]. Nevertheless, accurate results are achieved if taxonomic keys are updated regularly. Most keys are over 35 years old and do not correspond to intraspecies phenotypic plasticity although they are quite useful in initial species clustering. Moreover, the presence of subpopulations at early stages of genetic divergence, with no significant morphological changes compared to the main population, could be overlooked using traditional identification. This mechanism of speciation could lead to cryptic species unknown to date [22]. Furthermore, for many sand fly groups males or females, based on their morphology alone, can be impossible to identify [23]. For example, females of *P. similis* and *P. sergenti* are separated by comparing slight differences in the pharynx which can be confusing even for an experienced taxonomist since the keys that are used for morphological identification are based on type-species individuals. There are closely related species within the genus *Lutzomyia* whose females are indistinguishable, leading to the usage of wing morphometrics to solve these problems [24]. Wings of sand flies are, however, quite delicate and often get lost during field samplings.

Molecular identification can tackle identification problems. There are no prerequisites asked (i.e. gender, developmental stage) and can be fast and more reliable compared to morphology. DNA barcoding was created aiming to build a universal library of specific sequenced fragments of the mitochondrial gene that encodes the cytochrome *c* oxidase subunit 1 (*cox*1 or “barcode”) that will correspond to species, helping the scientific community to answer systematic questions [25]. In sand fly taxonomy research, DNA barcoding (i.e. identification via *cox*1 sequencing) is the second most used molecular identification method. It is quite popular in the New World and it advances rapidly in the Old World [23]. The method has helped to reveal cryptic sand fly species [26] and it has also been used to distinguish female sand flies between closely related species [22]. In Greece, recently, two different studies used the method to successfully identify sand flies in VL/CL/CanLei foci [27, 28].

This study presents the molecular identification of five wild caught *Phlebotomus* and *Sergentomyia* sand fly species from Crete and Cyprus based on DNA barcoding. A phylogenetic analysis, based on *cox*1 sequences, showed the systematic relationships of the sand flies caught with other sand flies circulating around the Mediterranean Basin. Furthermore, the publication of the sequences acquired through this study will help towards enriching the sand fly barcode library. Presence and possible vectorial capacity status of these medically important insects is discussed concerning the studied areas. Since DNA barcoding can save time and win on accuracy when compared to morphological identification, it could accompany traditional identification with morphological tools in order to verify questionable results. That way, possible mistakes or systematic discrepancies could be resolved and all individuals studied can be placed in their respective taxa.

## Methods

### Sand flies

All individuals were sampled during the EU EDENEXT project (FP7–261504) using methods already described [9]. After dissection of the insect bodies and morphological identification [29, 30], the remaining body parts were stored in 70% ethanol (Fisher Scientific, Schwerte, Germany) for molecular use. For this study, 31 sand flies of both genders, collected from the islands of Crete (Fodele: 35°22′52.10″N, 24°57′28.55″E) and the Republic of Cyprus (Geri: 35° 6′0.10″N, 33°25′17.40″E, Steni: 34°59′54.00″N, 32°28′17.00″E), were selected randomly representing five species belonging to four subgenera and two genera (Table 1).

**Table 1:** List of *Phlebotomus* and *Sergentomyia* sandflies studied

	Species	Gender	Collection site	Collection date	BLAST result (E-value)	GenBank ID
1	*Phlebotomus papatasi*	F	Cyprus (S)	October 2013	*P. papatasi* (0.0)	MF968973
2	*Phlebotomus papatasi*	F	Cyprus (G)	June 2011	*P. papatasi* (0.0)	MF968974
3	*Phlebotomus papatasi*	M	Cyprus (G)	June 2011	*P. papatasi* (0.0)	MF968970
4	*Phlebotomus papatasi*	M	Cyprus (S)	July 2013	*P. papatasi* (0.0)	MF968971
5	*Phlebotomus papatasi*	M	Cyprus (S)	July 2013	*P. papatasi* (0.0)	MF968972
6	*Phlebotomus papatasi*	F	Crete	October 2014	*P. papatasi* (0.0)	MF968980
7	*Phlebotomus papatasi*	F	Crete	June 2012	*P. papatasi* (0.0)	MF968989
8	*Phlebotomus papatasi*	F	Crete	June 2013	*P. papatasi* (0.0)	MF968991
9	*Phlebotomus papatasi*	F	Crete	July 2013	*P. papatasi* (0.0)	MF968992
10	*Phlebotomus papatasi*	M	Crete	May 2013	*P. papatasi* (0.0)	MF968993
11	*Phlebotomus papatasi*	M	Crete	June 2013	*P. papatasi* (0.0)	MF968990
12	*Phlebotomus similis*	F	Crete	June 2011	*P. sergenti* (0.0)^a^	MF968994
13	*Phlebotomus similis*	F	Crete	July 2011	*P. sergenti* (0.0)^a^	MF968995
14	*Phlebotomus similis*	F	Crete	June 2011	*P. sergenti* (0.0)^a^	MF968983
15	*Phlebotomus similis*	F	Crete	May 2012	*P. sergenti* (0.0)^a^	MF968984
16	*Phlebotomus similis*	M	Crete	June 2011	*P. sergenti* (0.0)^a^	MF968982
17	*Phlebotomus similis*	M	Crete	July 2014	*P. sergenti* (0.0)^a^	MF968985
18	*Phlebotomus killicki*	F	Cyprus (S)	March 2014	*P. killicki* (0.0)	MF968978
19	*Phlebotomus killicki*	M	Cyprus (S)	March 2014	*P. killicki* (0.0)	MF968979
20	*Sergentomyia minuta*	F	Crete	July 2013	*S. minuta* (0.0)	MF968997
21	*Sergentomyia minuta*	F	Cyprus (S)	July 2013	*S. minuta* (0.0)	MF968996
22	*Sergentomyia minuta*	M	Cyprus (S)	July 2013	*S. minuta* (0.0)	MF968988
23	*Sergentomyia minuta*	M	Cyprus (S)	June 2013	*S. minuta* (0.0)	MF968986
24	*Sergentomyia minuta*	M	Cyprus (S)	June 2013	*S. minuta* (0.0)	MF968981
25	*Sergentomyia minuta*	M	Cyprus (S)	July 2013	*S. minuta* (0.0)	MF969000
26	*Sergentomyia dentata*	F	Cyprus (S)	July 2012	*S. dentata* (0.0)	MF968999
27	*Sergentomyia dentata*	F	Cyprus (S)	June 2013	*S. dentata* (0.0)	MF968975
28	*Sergentomyia dentata*	F	Cyprus (S)	May 2013	*S. dentata* (0.0)	MF968987
29	*Sergentomyia dentata*	M	Cyprus (S)	May 2013	*S. dentata* (0.0)	MF968976
30	*Sergentomyia dentata*	M	Cyprus (S)	July 2013	*S. dentata* (0.0)	MF968977
31	*Sergentomyia dentata*	M	Cyprus (S)	May 2013	*S. dentata* (0.0)	MF968998

^a^There were no *P. similis cox*1 sequences in NCBI Nucleotide Database at the time of query (July 2017)

*Abbreviations: F* female, *M* male, *G* Geri village, *S* Steni village

### Sand fly DNA extraction, PCR and sequencing

The Qiagen QIAamp DNA micro kit (Qiagen, Hilden, Germany) was used to extract sand fly DNA. Barcoding region of *cox*1 gene was amplified using primers LCO1490/HCO2198 [31] under previously described conditions [25, 32]. PCR products resulting from *cox*1 amplification were purified using Qiagen QIAquick PCR Purification kit. PCR primers were used in double stranded sequencing which was performed in CEMIA SA (Larisa, Greece). Sequencing results quality was checked by eye and identity of all sequences was confirmed by BLAST™ queries. CodonCode Aligner™ (v. 3.7.1 CodonCode Corporation (Centerville, MA, USA) software was used for editing the sequences.

### Dataset

A dataset was created and used for phylogenetic analyses which included a total of 108 sequences. The set was comprised of the 31 *cox*1 sequences from the present study, 31 *Larroussius cox*1 sequences from Crete and Cyprus [27], 45 *cox*1 sequences of related sand fly taxa derived from GenBank™ and one *Culex pipiens cox*1 sequence as outgroup, also derived from GenBank™. The sequences were translated into amino acids using MEGA 7.0 [33] and no stop codons were observed. Multiple sequence alignments were performed using CLUSTALW [34] as implemented in MEGA. Genetic distances were calculated using Tamura-Nei model [35], also in MEGA.

### Phylogenetic analyses

The optimal partitioning scheme (unpartition or codon partition) and the best-fit nucleotide substitution model for each partition were identified using the Partition Finder (PF) v.1.1.1 [36]. PF was ran two different times with the models of molecular evolution restricted to those that are available in either MrBayes or RAxML, using the greedy search algorithm, linked branch lengths in calculations of likelihood scores, and the Bayesian information criterion (BIC) for selecting among alternative partitioning strategies. The models that include both a parameter for among-site rate heterogeneity (G) and a parameter for invariant sites (I) were ignored, because the adding of a proportion of invariable sites creates a strong correlation, making it impossible to estimate both parameters reliably. Another drawback of the model is that the estimate of the propotion of invariable sites (p0) is very sensitive to the number and divergences of the sequences included in the data [37]. Phylogenetic inference analyses were conducted using Bayesian inference (BI), and maximum likelihood (ML) methods.

The BI analysis was performed in MrBayes (v.3.2.6; [38]) with four runs and eight chains per run for 10^7^ generations sampling every 100th generation. This generated an output of 10^5^ trees. Several MCMC convergence diagnostics were used to check for convergence and stationarity. The first 25% of the trees (25% “burn-in” in Bayesian terms) were discarded as a measure to sample from the stationary distribution and avoid the possibility of including random, suboptimal trees. A majority rule consensus Bayesian tree was then calculated from the posterior distribution of trees, and the posterior probabilities were calculated as the percentage of samples recovering any particular clade [39], where probabilities higher than 95% were considered indicative of significant support.

ML analyses were conducted with RAxML v.8.1.21 [40] using RAxMLGUI v.1.5 [41] under the models selected in PF analyses where parameters were estimated independently for each partition. The best ML tree was selected from 500 iterations and the confidence of the branches of the best ML tree was assessed based on 1000 thorough bootstrap replicates.

## Results

### Identification of wild-caught sand flies

Thirty one sand flies (16 females and 15 males) from Crete and Cyprus were identified by morphology: 11 individuals as *P. papatasi*, 6 as *P. similis*, 2 as *P. killicki*, 6 as *S. minuta* and 6 as *S. dentata*. No intraspecies peculiarities were observed among the individuals examined. DNA barcoding resulted in *cox*1 sequences for each sample and BLAST™ queries confirmed their identity (Table 1).

### Sequence analysis

The dataset contained 636 bp of *cox*1 sequences. Variable sites were 245 while parsimony informative sites were 217. The pairwise genetic distances ranged from 0 to 25%. After grouping obtained sequences, according to species (Table 2), *Phlebotomus* spp. individuals had mean genetic distance between 6 and 21% while members of the genus *Sergentomyia* had less extreme distance range (13–17%). The highest intraspecific mean genetic distance (intraspecies delimitation), based on our dataset, was set as 3%. This value was calculated among the *S. minuta* individuals analyzed. On the other hand, the respected lowest interspecific value (interspecies delimitation) was set as 6%. This was assessed when calculating the distance between *P. syriacus* and its sister species *P. neglectus*. *Phlebotomus papatasi* sequences from Crete had 1% mean genetic distance from the ones from Cyprus. As for the *S. minuta* sequences, there was a 4% mean genetic distance between the sand flies from Greece and Cyprus and a 3.3% between *S. minuta* sequences from Crete and mainland Greece. Intraspecies mean genetic distance of all *S. minuta* sequences, regardless their origin, was less than 1%. The analysis of PF supported the partitioning of the dataset in the three codon positions. The nucleotide substitution model selected for each data partition were: for MrBayes SYM + I for the first codon position, HKY + I for the second and GTR + G for the third codon position, whereas GTR + G for each one of the codon positions for RAxML.

**Table 2:** Among species genetic distances (in %) based on the Tamura-Nei model. Diagonal line in bold shows intraspecies distances

		1	2	3	4	5	6	7	8	9	10	11
1	*P. papatasi*	**1**										
2	*P. similis*	18	**2**									
3	*S. dentata*	20	20	**2**								
4	*P. tobbi*	14	19	18	**1**							
5	*S. minuta*	18	20	17	18	**3** ^**a**^						
6	*P. perfiliewi*	14	21	17	8	16	**2**					
7	*P. neglectus*	16	21	21	14	18	13	**0**				
8	*P. killicki*	12	16	18	13	16	12	16	**1**			
9	*P. syriacus*	12	18	20	13	17	11	**6** ^**a**^	13	**na**		
10	*P. sergenti*	15	12	18	12	18	16	16	16	15	**0**	
11	*S. fallax*	17	19	13	14	17	18	16	18	16	16	**1**

*Abbreviation: na* not applicable

^a^Indicate the high intraspecific and low interspecific distances, respectively, based on the dataset

### Phylogenetic analyses

**Fig. 1:**
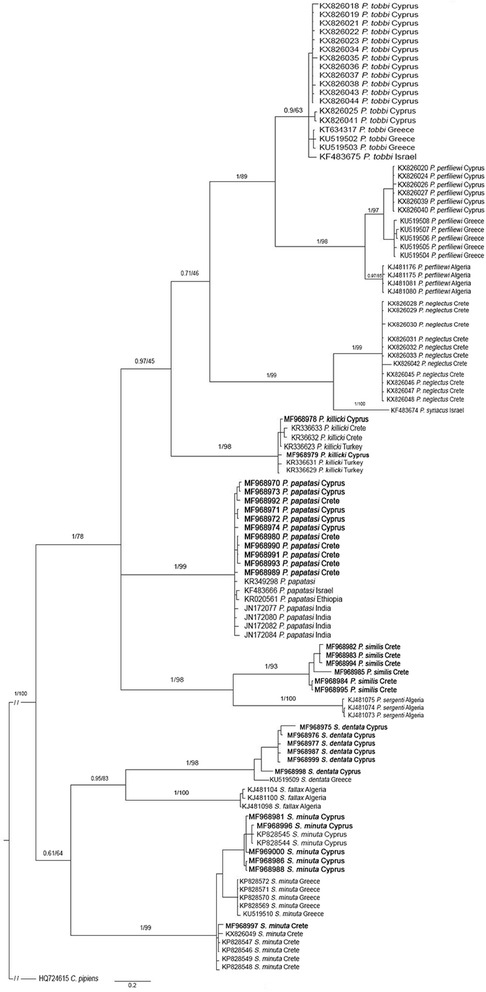
Bayesian inference (BI) tree (number above branches represent BI posterior probabilities and bootstrap support from a maximum likelihood (ML) analysis as BI/ML). *Culex pipiens* was used as the outgroup. Species names correspond to Table 1. Individuals from the present study appear in bold

Maximum likelihood (-lnL = 4340.76), and Bayesian inference analysis (arithmetic mean -lnL = 4352.85) produced similar topologies. Considering the Bayesian analysis, the MCMC convergence diagnostics (average standard deviation of split frequencies, the plot of the generation versus the log probability of the data, the average Potential Scale Reduction Factor, and the minimum value of minimum Estimated Sample Sizes) revealed no clues of non-convergence, indicating stationarity, that is, there should be no tendency of increase or decrease over time. The Bayesian phylogenetic tree presented in Fig. 1 demonstrates a clear separation between the two genera, *Phlebotomus* and *Sergentomyia*, and also the subgenera *Phlebotomus*, *Paraphlebotomus*, *Transphlebotomus* and *Sergentomyia*, respectively.

All species were separated with each one having its own branch. Sequences for all newly collected individuals clustered together with those already published for the respective species of the same or different locality forming groups of the same subgenus. The phylogenetic groupings provided by the tree, coupled with the aforementioned sequencing queries against GenBank™, confirmed the molecular and morphological identification of the sampled sand flies.

## Discussion

The present study used DNA barcoding to identify 31 wild-caught sand flies, belonging to five species, from Crete and Cyprus. The species identified are *P. papatasi* (from both Crete and Cyprus), *P. similis* (from Crete), *P. killicki* (from Cyprus), *S. minuta* (from both Crete and Cyprus) and *S. dentata* (from Cyprus). After obtaining the *cox*1 sequences necessary for DNA barcoding, a larger dataset of *cox*1 barcodes was constructed in order to place the new ones in a phylogenetic tree that would describe the relationships between sand fly species around the Mediterranean Basin and conclusively verify their identity (Fig. 1).

*Phlebotomus papatasi* is the vector of *L. major* that causes zoonotic CL in humans in many countries in Africa and Asia [42]. It can be found locally all around southern Europe but there are no studies of its vectorial role in Greece [12]. Given that *P. papatasi* has an established presence in Crete and mainland Greece [4, 7, 8, 43, 44] as well as in Cyprus [14, 18], population monitoring should continue to determine whether it can act as the vector of *L. major* in these areas in case infected rodent reservoirs are introduced. In the present study, *P. papatasi* individuals were well separated with high posterior probability (Fig. 1) from other species groups and clustered together with published *cox*1 barcodes of the same species. *Phlebotomus papatasi* belongs to the phylogenetically distinct subgenus *Phlebotomus*, members of which have been quite often used in phylogenetic analyses [18, 28, 45, 46]. In a study where 22 populations of *P. papatasi* from 16 countries were subjected to phylogenetic analysis, it was shown that samples from Crete and Cyprus shared haplotypes implying close genetic relationships albeit their insular isolation [47]. The present study encountered two individuals that share a haplotype (data not shown). This fact, along with the 1% mean genetic distance between the samples of *P. papatasi* from Greece and Cyprus of the present study, adds more supporting information to that conclusion. Additionally, their clear morphological homogeneity suggests that these two populations, most likely, do not constitute different taxa. All individuals analyzed in the present study represented a single clade although they were derived from quite diverse geographical locations (India, Ethiopia, Israel). This observation indicates that more studies are needed to investigate the possibility of cryptic or sibling taxa within this geographically diverse species group.

*Phlebotomus similis* is the sister species of *P. sergenti,* a proven vector of *L. tropica* [11, 48, 49]. Due to its abundance in CL foci in Greece, it is believed that this is the species transmitting *L. tropica* in the country [50]. Sand fly samplings in Greece, in the past, reported the presence of *P. sergenti*. However, Depaquit et al. [11] suggested that the species found is actually *P. similis* and that this is the sole *Paraphlebotomus* species found in the country able to transmit *L. tropica*. In fact, since 2002, no published sand fly samplings in Greece have reported *P. sergenti* [7, 16, 27]. Additionally, sequencing analysis showed that *P. similis* individuals from this study exhibit a 12% distance from *P. sergenti* sequences derived from Algeria, separating these species in a clear manner. This is the first time that *cox*1 sequences of *P. similis* are deposited in GenBank, a first step to start monitoring *P. similis* populations to resolve their systematic status via DNA barcoding. Molecular studies of the subgenus *Paraphlebotomus* based on nuclear [51] and mitochondrial markers [45, 46] have come to same topology conclusions as the present study.

*Phlebotomus killicki* was recently described as a member of the subgenus *Transphlebotomus* and it was found in locations in Crete and Turkey, sites 500 km apart, along with *P. anatolicus* [30]. In Cyprus, the presence of *P. economidesi* was reported along with that of *P. mascittii* [14, 52] but its presence on the island should be re-evaluated [30]. *Phlebotomus economidesi* was also found in Turkey [30]. This is the first report of *P. killicki* in Cyprus; where this species was found in sympatry with *P. economidesi*. However, further samplings will determine whether there are other sympatry phenomena, as for example with *P. mascittii* [30]. As for the phylogenetic relationships between the available *P. killicki* individuals, the samples from Cyprus were not separated from those collected in Turkey or Crete [30]. Another *Transphlebotomus* species, *P. canaaniticus*, occurs in the Middle East [53]. *Phlebotomus mascittii* is the *Transphlebotomus* species presenting the widest geographical distribution compared with other species within that subgenus (from Germany and Austria [54, 55] to Crete [4, 7]). *Transphlebotomus* is a subgenus that is closely related to the subgenera *Adlerius* and *Larroussius* [56] which both contain *Leishmania* vectors [57] and have being thoroughly studied [27, 58–60]. It is suggested that since populations of *Transphlebotomus* spp. sand flies are detected more often than before, more studies for their vectorial role should be conducted.

*Sergentomyia minuta* and *S. dentata* are known to be present in mainland Greece [7, 44] as well as in the islands of Crete [6–8] and Cyprus [14, 18]. *Sergentomyia minuta* has a doubtful taxonomic status and a quite unique intraspecific variability [19] which should be evaluated together with its possible vectorial capacity. All *Sergentomyia* samples sequenced in this work clustered by genus, species and locality, respectively, confirming previous studies [28, 61]. The *S. minuta* clade (Fig. 1) presented a geographical locality-based separation and the three sub-clades created suggest that speciation events could be underway. Genetic distances between the three sub-clades (Cyprus, mainland Greece, Crete) exceed 3% while all three intra-clade distances did not exceed 1%, evidence that supports the above-mentioned isolation based on this dataset. Of particular note, *Sergentomyia* sp. individuals were caught using sticky traps [9], mostly among rock pile wall formations which provide resting and hiding places for lizards. This genus contains sand fly species that feed on reptiles and transmit *Sauroleishmania*, a parasite infecting reptiles [10]. Such rock formations also provide a perfect nesting habitat for sand flies. Since sand flies are known to be weak flyers, they tend to reside close to their blood meal sources [1]. Given that, it is suggested that *S. minuta* populations, in their specific habitats, may have been isolating themselves faster than other species thus creating such an intraspecies diversity as the one demonstrated here (Fig. 1). As this evidence underlines the possible existence of cryptic taxa, further samplings for individuals in those areas are being conducted. The genetic isolation between individuals from Crete and Cyprus is not supported with morphological findings indicating that further multigene molecular work, accompanied with morphospecies clustering, would provide further information on this interesting issue. There are an increasing number of studies that detect *Leishmania* DNA in sand flies belonging to the genus *Sergentomyia* [62–65] but detection of parasite DNA in sand flies is not sufficient evidence for a species to be classified as vector [66]. A study in Senegal examined whether *Sergentomyia* sand fly species were vectors of *L. infantum* in a CanLei endemic focus where *Phlebotomus* species are absent or significantly under-represented. It was concluded that *S. dubia* and *S. schwetzi* are the possible vectors of the parasite in the studied area [67] based on accepted vectorial criteria [68]. As a result, it appears that *Sergentomyia* needs to be studied more thoroughly since there are more taxonomic and vectorial capacity questions requiring answers [19, 61].

## Conclusions

This study constitutes the molecular identification of the sand fly species caught in two VL/CL/CanLei foci, Cyprus and Crete, in the southeastern Mediterranean, using DNA barcoding. It makes a contribution towards understanding the systematic status of *P. similis*, the suspected vector of *L. tropica* in Greece. This is the first time DNA barcoding has been applied to this important species and the derived barcodes are added to the GenBank database. The presence of *P. killicki* is reported for the first time in Cyprus and possible newly arising taxa within the *S. minuta* phylogenetic clade are demonstrated. Regarding the *P. papatasi* individuals caught in the two islands, it was shown that although the two populations are geographically unassociated, they show no morphological or DNA barcoding-based differences. As more barcodes are added to the database, identification/clustering process of sand flies and their molecular systematics will be accurately resolved. Since sand fly species are quite important in the transmission of *Leishmania* parasites as well as other pathogens, their geographical distribution and vectorial capacity must be extensively evaluated. DNA barcoding helps towards that direction by putting the stepping stone to the comprehension of taxa.

## Abbreviations

BI: Bayesian inference
BIC: Bayesian information criterion
CanLei: Canine leishmaniasis
CL: Cutaneous leishmaniasis
*cox*1: Cytochrome oxidase I
EU: European Union
ML: Maximum likelihood
PCR: Polymerase chain reaction
PF: Partition finder
VL: Visceral leishmaniasis

## References

[1] Killick-Kendrick R (1999). The biology and control of phlebotomine sand flies. Clin Dermatol.

[2] World Health Organization (WHO). 2017. http://www.who.int/leishmaniasis/en/. Accessed Jul 2017.

[3] Killick-Kendrick R. Some epidemiological consequences of the evolutionary fit between leishmaniae and their phlebotomine vectors. Bull Soc Pathol Exot Filiales. 1985;78(5 Pt 2):747–55. 3836763

[4] Christodoulou V, Antoniou M, Ntais P, Messaritakis I, Ivovic V, Dedet J-P (2012). Re-emergence of visceral and cutaneous leishmaniasis in the Greek Island of Crete. Vector Borne Zoonotic Dis.

[5] Léger N, Gramiccia M, Gradoni L, Madulo-Leblond G, Pesson B, Ferté H, et al. Isolation and typing of *Leishmania infantum* from *Phlebotomus neglectus* on the Island of Corfu, Greece. Trans R Soc Trop Med Hyg. 1988;82(3):419–20. 10.1016/0035-9203(88)90145-9 3232178

[6] Léger N, Pesson B, Madulo-Leblond G, Ferte H, Tselentis Y, Antoniou M (1993). The phlebotomes of Crete. Biologia Gallo-Hellenica.

[7] Ivović V, Patakakis M, Tselentis Y, Chaniotis B (2007). Faunistic study of sandflies in Greece. Med Vet Entomol.

[8] Ntais P, Christodoulou V, Tsirigotakis N, Dokianakis E, Dedet J-P, Pratlong F, et al. Will the introduction of *Leishmania tropica* MON-58, in the Island of Crete, lead to the settlement and spread of this rare zymodeme? Acta Trop. 2014;132:125–30. 10.1016/j.actatropica.2014.01.003 24462941

[9] Alten B, Maia C, Afonso MO, Campino L, Jiménez M, González E, et al. Seasonal dynamics of phlebotomine sand fly species proven vectors of Mediterranean leishmaniasis caused by *Leishmania infantum*. PLoS Negl Trop Dis. 2016;10(2):e0004458. 10.1371/journal.pntd.0004458 PMC4762948 26900688

[10] Killick-Kendrick R (1990). Phlebotomine vectors of the leishmaniases: a review. Med Vet Entomol.

[11] Depaquit J, Ferté H, Léger N, Lefranc F, Alves-Pires C, Hanafi H (2002). ITS 2 sequences heterogeneity in *Phlebotomus sergenti* and *Phlebotomus similis* (Diptera, Psychodidae): possible consequences in their ability to transmit *Leishmania tropica*. Int J Parasitol.

[12] Ready PD (2010). Leishmaniasis emergence in Europe. Euro Surveill.

[13] Léger N, Depaquit J, Ferté H, Rioux JA, Gantier JC, Gramiccia M (2000). Phlebotomine sandflies (Diptera-Psychodidae) of the isle of Cyprus. II-isolation and typing of *Leishmania* (*Leishmania infantum*) Nicolle, 1908 (zymodeme MON 1) from *Phlebotomus* (*Larroussius*) *tobbi* Adler & Theodor, 1930. Parasite.

[14] Mazeris A, Soteriadou K, Dedet JP, Haralambous C, Tsatsaris A, Moschandreas J (2010). Leishmaniases and the Cyprus paradox. Am J Trop Med Hyg..

[15] Antoniou M, Haralambous C, Mazeris A, Pratlong F, Dedet J-P, Soteriadou K (2008). *Leishmania donovani* leishmaniasis in Cyprus. Lancet Infect Dis.

[16] Antoniou M, Haralambous C, Mazeris A, Pratlong F, Dedet J-P, Soteriadou K (2009). *Leishmania donovani* leishmaniasis in Cyprus. Lancet Infect Dis.

[17] Ntais P, Sifaki-Pistola D, Christodoulou V, Messaritakis I, Pratlong F, Poupalos G (2013). Leishmaniases in Greece. Am J Trop Med Hyg..

[18] Ergunay K, Kasap OE, Orsten S, Oter K, Gunay F, Yoldar AZA (2014). *Phlebovirus* and *Leishmania* detection in sandflies from eastern Thrace and northern Cyprus. Parasit Vectors.

[19] Depaquit J, Hadj-Henni L, Bounamous A, Strutz S, Boussaa S, Morillas-Marquez F (2015). Mitochondrial DNA intraspecific variability in *Sergentomyia minuta* (Diptera: Psychodidae). J Med Entomol.

[20] Jaouadi K, Haouas N, Chaara D, Boudabous R, Gorcii M, Kidar A (2013). Phlebotomine (Diptera, Psychodidae) bloodmeal sources in tunisian cutaneous leishmaniasis foci: could *Sergentomyia minuta*, which is not an exclusive herpetophilic species, be implicated in the transmission of pathogens?. Ann Entomol Soc Am.

[21] Lewis DJ (1978). Phlebotomid sand flies (Diptera: Psychodidae) of the oriental region. Bull World Health Organ.

[22] Pinto I de S, das CBD, Rodrigues AAF, Ferreira AL, Rezende HR, Bruno RV (2015). DNA barcoding of Neotropical sand flies (Diptera, Psychodidae, Phlebotominae): species identification and discovery within Brazil. PLoS One.

[23] Depaquit J. Molecular systematics applied to phlebotomine sandflies: review and perspectives. Infect Genet Evol. 2014;28:744–756. 10.1016/j.meegid.2014.10.027 25445650

[24] Giordani BF, Andrade AJ, Galati EAB, Gurgel-Gonçalves R (2017). The role of wing geometric morphometrics in the identification of sandflies within the subgenus *Lutzomyia*. Med Vet Entomol.

[25] Hebert PDN, Cywinska A, Ball SL, deWaard JR (2003). Biological identifications through DNA barcodes. Proc Biol Sci.

[26] Gajapathy K, Tharmasegaram T, Eswaramohan T, LBSL P, Jayanetti R, Surendran SN (2016). DNA barcoding of Sri Lankan phlebotomine sand flies using cytochrome c oxidase subunit I reveals the presence of cryptic species. Acta Trop.

[27] Dokianakis E, Tsirigotakis N, Christodoulou V, Poulakakis N, Antoniou M (2016). DNA sequencing confirms PCR-RFLP identification of wild-caught *Larroussius* sand flies from Crete and Cyprus. Acta Trop.

[28] Chaskopoulou A, Giantsis IA, Demir S, Bon MC (2016). Species composition, activity patterns and blood meal analysis of sand fly populations (Diptera: Psychodidae) in the metropolitan region of Thessaloniki, an endemic focus of canine leishmaniasis. Acta Trop.

[29] Lewis DJ (1982). A taxonomic review of the genus *Phlebotomus* (Diptera: Psychodidae). Bull Br Mus Nat Hist Entomol.

[30] Kasap OE, Dvorak V, Depaquit J, Alten B, Votypka J, Volf P. Phylogeography of the subgenus *Transphlebotomus* Artemiev with description of two new species, *Phlebotomus anatolicus* n. Sp. and *Phlebotomus killicki* n. sp. Infect Genet Evol. 2015;34:467–79. 10.1016/j.meegid.2015.05.025 26006062

[31] Folmer O, Black M, Hoeh W, Lutz R, Vrijenhoek R. DNA primers for amplification of mitochondrial cytochrome c oxidase subunit I from diverse metazoan invertebrates. Mol Marine Biol Biotechnol. 1994;3(5):294–9. 7881515

[32] Cohnstaedt LW, Beati L, Caceres AG, Ferro C, Munstermann LE (2011). Phylogenetics of the phlebotomine sand fly group Verrucarum (Diptera: Psychodidae: *Lutzomyia*). Am J Trop Med Hyg.

[33] Kumar S, Stecher G, Tamura K (2016). MEGA7: molecular evolutionary genetics analysis version 7.0 for bigger datasets. Mol Biol Evol.

[34] Thompson JD, Higgins DG, Gibson TJ (1994). CLUSTAL W: improving the sensitivity of progressive multiple sequence alignment through sequence weighting, position-specific gap penalties and weight matrix choice. Nucleic Acids Res.

[35] Tamura K, Nei M (1993). Estimation of the number of nucleotide substitutions in the control region of mitochondrial DNA in humans and chimpanzees. Mol Biol Evol.

[36] Lanfear R, Calcott B, Ho SYW, Guindon S (2012). Partitionfinder: combined selection of partitioning schemes and substitution models for phylogenetic analyses. Mol Biol Evol.

[37] Yang Z (2016). Computational Molecular Evolution.

[38] Ronquist F, Teslenko M, van der Mark P, Ayres DL, Darling A, Höhna S (2012). MrBayes 3.2: efficient Bayesian phylogenetic inference and model choice across a large model space. Syst Biol.

[39] Huelsenbeck JP, Ronquist F (2001). MRBAYES: Bayesian inference of phylogenetic trees. Bioinformatics.

[40] Stamatakis A (2014). RAxML version 8: a tool for phylogenetic analysis and post-analysis of large phylogenies. Bioinformatics.

[41] Silvestro D, Michalak I (2011). raxmlGUI: a graphical front-end for RAxML. Org Divers Evol.

[42] Maroli M, Feliciangeli MD, Bichaud L, Charrel RN, Gradoni L (2013). Phlebotomine sandflies and the spreading of leishmaniases and other diseases of public health concern. Med Vet Entomol.

[43] Depaquit J, Grandadam M, Fouque F, Andry PE, Peyrefitte C (2010). Arthropod-borne viruses transmitted by phlebotomine sandflies in Europe: a review. Euro Surveill..

[44] Xanthopoulou K, Anagnostou V, Ivovic V, Djurkovic-Djakovic O, Rogozi E, Sotiraki S (2011). Distribution of sandflies (Diptera, Psychodidae) in two Ionian Islands and northern Greece. Vector Borne Zoonotic Dis..

[45] Bounamous A, Lehrter V, Hadj-Henni L, Delecolle J-C, Depaquit J (2014). Limits of a rapid identification of common Mediterranean sandflies using polymerase chain reaction-restriction fragment length polymorphism. Mem Inst Oswaldo Cruz.

[46] Maia C, Parreira R, Cristóvão JM, Afonso MO, Campino L (2015). Exploring the utility of phylogenetic analysis of cytochrome oxidase gene subunit I as a complementary tool to classical taxonomical identification of phlebotomine sand fly species (Diptera, Psychodidae) from southern Europe. Acta Trop.

[47] Depaquit J, Lienard E, Verzeaux-Griffon A, Ferté H, Bounamous A, Gantier J-C (2008). Molecular homogeneity in diverse geographical populations of *Phlebotomus papatasi* (Diptera, Psychodidae) inferred from ND4 mtDNA and ITS2 rDNA. Epidemiological consequences. Infect Genet Evol.

[48] Volf P, Ozbel Y, Akkafa F, Svobodová M, Votýpka J, Chang KP (2002). Sand flies (Diptera: Phlebotominae) in Sanliurfa, Turkey: relationship of *Phlebotomus sergenti* with the epidemic of anthroponotic cutaneous leishmaniasis. J Med Entomol.

[49] Ajaoud M, Es-sette N, Hamdi S, El-Idrissi AL, Riyad M, Lemrani M (2013). Detection and molecular typing of *Leishmania tropica* from *Phlebotomus sergenti* and lesions of cutaneous leishmaniasis in an emerging focus of Morocco. Parasit Vectors.

[50] Antoniou M, Gramiccia M, Molina R, Dvorak V, Volf P (2013). The role of indigenous phlebotomine sandflies and mammals in the spreading of leishmaniasis agents in the Mediterranean region. Euro Surveill..

[51] Depaquit J, Ferté H, Léger N, Killick-Kendrick R, Rioux JA, Killick-Kendrick M (2000). Molecular systematics of the phlebotomine sandflies of the subgenus *Paraphlebotomus* (Diptera, Psychodidae, *Phlebotomus*) based on ITS2 rDNA sequences. Hypotheses of dispersion and speciation. Insect Mol Biol.

[52] Léger N, Depaquit J, Ferté H. Phlebotomine sandflies (Diptera-Psychodidae) of the isle of Cyprus. I-description of *Phlebotomus* (*Transphlebotomus*) *economidesi* n. sp. Parasite. 2000;7(2):135–41. 10.1051/parasite/2000072135 10887661

[53] Sawalha SS, Shtayeh MS, Khanfar HM, Warburg A, Abdeen ZA. Phlebotomine sand flies (Diptera: Psychodidae) of the Palestinian West Bank: potential vectors of leishmaniasis. J Med Entomol. 2003;40(3):321–8. 10.1603/0022-2585-40.3.321 12943111

[54] Melaun C, Krüger A, Werblow A, Klimpel S (2014). New record of the suspected leishmaniasis vector *Phlebotomus* (*Transphlebotomus*) *mascittii* Grassi, 1908 (Diptera: Psychodidae: Phlebotominae) - the northernmost phlebotomine sandfly occurrence in the Palearctic region. Parasitol Res.

[55] Obwaller AG, Karakus M, Poeppl W, Töz S, Özbel Y, Aspöck H (2016). Could *Phlebotomus mascittii* play a role as a natural vector for *Leishmania infantum*? New data. Parasit Vectors.

[56] Depaquit J, Naucke TJ, Schmitt C, Ferté H, Léger N (2005). A molecular analysis of the subgenus *Transphlebotomus* Artemiev, 1984 (*Phlebotomus*, Diptera, Psychodidae) inferred from ND4 mtDNA with new northern records of *Phlebotomus mascittii* Grassi, 1908. Parasitol Res.

[57] World Health Organization (WHO). Control of the leishmaniases. World Health Organ Tech Rep. 2010;Ser xii–xiii:1–186. 21485694

[58] Di Muccio T, Marinucci M, Frusteri L, Maroli M, Pesson B, Gramiccia M (2000). Phylogenetic analysis of *Phlebotomus* species belonging to the subgenus *Larroussius* (Diptera, Psychodidae) by ITS2 rDNA sequences. Insect Biochem Mol Biol.

[59] Depaquit J, Bounamous A, Akhoundi M, Augot D, Sauvage F, Dvorak V (2013). A taxonomic study of *Phlebotomus* (*Larroussius*) *perfiliewi s.l*. Infect Genet Evol.

[60] Zahraei-Ramazani A, Kumar D, Mirhendi H, Sundar S, Mishra R, Moin-Vaziri V (2015). Morphological and genotypic variations among the species of the subgenus *Adlerius* (Diptera: Psychodidae, *Phlebotomus*) in Iran. J Arthropod Borne Dis.

[61] Maia C, Depaquit J (2016). Can *Sergentomyia* (Diptera, Psychodidae) play a role in the transmission of mammal-infecting *Leishmania*?. Parasite.

[62] Berdjane-Brouk Z, Koné AK, Djimdé AA, Charrel RN, Ravel C, Delaunay P (2012). First detection of *Leishmania major* DNA in *Sergentomyia* (*Spelaeomyia*) *darlingi* from cutaneous leishmaniasis foci in Mali. PLoS One.

[63] Campino L, Cortes S, Dionísio L, Neto L, Afonso MO, Maia C (2013). The first detection of *Leishmania major* in naturally infected *Sergentomyia minuta* in Portugal. Mem Inst Oswaldo Cruz.

[64] Nzelu CO, Kato H, Puplampu N, Desewu K, Odoom S, Wilson MD (2014). First detection of *Leishmania tropica* DNA and *Trypanosoma* species in *Sergentomyia* sand flies (Diptera: Psychodidae) from an outbreak area of cutaneous leishmaniasis in Ghana. PLoS Negl Trop Dis.

[65] Jaouadi K, Ghawar W, Salem S, Gharbi M, Bettaieb J, Yazidi R (2015). First report of naturally infected *Sergentomyia minuta* with *Leishmania major* in Tunisia. Parasit Vectors.

[66] Seblova V, Sadlova J, Carpenter S, Volf P (2014). Speculations on biting midges and other bloodsucking arthropods as alternative vectors of *Leishmania*. Parasit Vectors.

[67] Senghor MW, Niang AA, Depaquit J, Ferté H, Faye MN, Elguero E (2016). Transmission of *Leishmania infantum* in the canine leishmaniasis focus of Mont-Rolland, Senegal: ecological, parasitological and molecular evidence for a possible role of *Sergentomyia* sand flies. PLoS Negl Trop Dis.

[68] Ready PD (2013). Biology of phlebotomine sand flies as vectors of disease agents. Annu Rev Entomol.

